# PuFFIN - a parameter-free method to build nucleosome maps from paired-end reads

**DOI:** 10.1186/1471-2105-15-S9-S11

**Published:** 2014-09-10

**Authors:** Anton Polishko, Evelien M Bunnik, Karine G Le Roch, Stefano Lonardi

**Affiliations:** 1 Department of Computer Science and Engineering, University of California, Riverside, CA 92521, US; 2 Department of Cell Biology and Neuroscience, University of California, Riverside CA 92521, US

**Keywords:** Nucleosome positioning, genome-wide nucleosome maps, paired-end reads, ChIP-Seq, MNase-Seq

## Abstract

**Background:**

We introduce a novel method, called PuFFIN, that takes advantage of paired-end short reads to build genome-wide nucleosome maps with larger numbers of detected nucleosomes and higher accuracy than existing tools. In contrast to other approaches that require users to optimize several parameters according to their data (e.g., the maximum allowed nucleosome overlap or legal ranges for the fragment sizes) our algorithm can accurately determine a genome-wide set of non-overlapping nucleosomes without any user-defined parameter. This feature makes PuFFIN significantly easier to use and prevents users from choosing the "wrong" parameters and obtain sub-optimal nucleosome maps.

**Results:**

PuFFIN builds genome-wide nucleosome maps using a multi-scale (or multi-resolution) approach. Our algorithm relies on a set of nucleosome "landscape" functions at different resolution levels: each function represents the likelihood of each genomic location to be occupied by a nucleosome for a particular value of the smoothing parameter. After a set of candidate nucleosomes is computed for each function, PuFFIN produces a consensus set that satisfies non-overlapping constraints and maximizes the number of nucleosomes.

**Conclusions:**

We report comprehensive experimental results that compares PuFFIN with recently published tools (NOrMAL, TEMPLATE FILTERING, and NucPosSimulator) on several synthetic datasets as well as real data for *S. cerevisiae *and *P. falciparum*. Experimental results show that our approach produces more accurate nucleosome maps with a higher number of non-overlapping nucleosomes than other tools.

## Background

One of the central objectives in molecular biology is to characterize all cellular processes controlling gene regulation. The complex interaction between DNA chromatin structure and transcription factors is one of these key processes. The basic unit of chromatin structure is the *nucleosome*, which is composed of *≈ *146 base pairs of DNA wrapped around a protein complex of eight histones. Loosely speaking, the more compact the chromatin, the harder it is for transcription factors and other DNA binding proteins to access DNA and trigger transcription. Thus, to elucidate the role of interactions between chromatin and transcription factors, it is crucial to determine the location of all nucleosomes along the chromosomes.

Several experimental techniques are available to produce genome-wide nucleosome maps. For instance, one can isolate the portions of DNA that are free of nucleosomes or enrich for genomic regions that are bound to histones. The latter can be achieved via micrococcal nuclease digestion (MNase or MAINE) [1], which can be combined with chromatin immunoprecipitation (ChIP) to enrich for a particular subset of nucleosomes (e.g., for a particular histone tail mark), typically followed by high-throughput sequencing (MNase-Seq and ChIP-Seq, respectively). In this work, we assume that the sequencing data is either MNase-Seq or ChIP-Seq, which are currently the most popular approaches to study the locations of nucleosomes and histone modifications.

An analysis of the literature reveals that the majority of nucleosome maps have so far been produced from single-end reads (which are less expensive to obtain than paired-end reads). As a consequence, nearly all computational methods available assume that the input data are single-end reads. Nucleosome positioning from single-end reads is, however, more computationally challenging and much less precise than if paired-end data was available. Paired-end reads allow one to determine both ends of nucleosome-enriched DNA fragments, whereas with single-end reads one either obtains one "boundary" or the other. In the latter case, the problem of associating a peak in the forward strand with the correct peak in the negative strand can be difficult, in particular for complex nucleosome configurations.

Existing methods for single-end reads either rely on the assumption that nucleosome-enriched DNA fragments are expected to be of a size compatible with the nucleosome (*≈ *146 bp), or use probabilistic models to estimate these sizes from the data. From our experience, the first approach can lead to poor results because there is no fragment size that will work equally well for all nucleosomes in the genome. While one would expect nucleosome-enriched DNA fragments to be about 146 bp, in MNase-Seq the digestion process can either leave nucleosome-free DNA in the sample, or "over-digest" the ends of nucleosome-bound DNA. Furthermore, the rate of digestion is sequence-dependent [2,3], so nucleosomes in different genomic locations can end up with different DNA fragment sizes.

Despite these challenges, the majority of so-called "peak-calling" approaches usually rely on the assumption that the data is derived from nucleosome-sized DNA fragments and consist of following steps: (1) a nucleosome occupancy score function is obtained from mapping nucleosome-enriched reads to the reference genome, followed by counting, smoothing and normalization; (2) candidate nucleosomes are placed according to the peaks of the score function; (3) the final set of nucleosomes is selected to satisfy additional constraints (which are tool-dependent). To compute the occupancy score, different techniques have been proposed, ranging from simply computing the number of reads covering each genomic location, to sophisticated statistics to estimate the false discovery rate. For instance, nucleR [4] uses the raw coverage with extensive "profile cleaning" based on the Fourier transform, whereas NSeq [5] employs a triangle statistic based on read counts within a sliding window.

A second group of methods is based on probabilistic models. Our tool NOrMAL [6] uses a modified Gaussian mixture model to infer nucleosome-enriched fragment sizes. The parametric probabilistic model allows to deal with the problem of overlapping and complex configurations of nucleosomes. Developed in parallel with NOrMAL, Ping [7] employs a similar probabilistic model. Both tools provide a clear advantage over algorithms that rely on the user to provide estimated DNA fragment sizes.

Finally, a distinct group of positioning methods depend on the availability of a control track (i.e., "naked" DNA), e.g., NucleoFinder [8], while others have been designed to perform differential nucleosome positioning, e.g., DANPOS [9] and DiNuP [10].

In this work, we focus on the problem of determining nucleosome positions based on the availability of paired-end reads (without a control track). To the best of our knowledge, NucPosSimulator [11] is the only published tool specifically designed to take advantage of paired-end reads: to place nucleosomes it solves the optimization problem of selecting the subset of peaks which maximizes the total score, under the constraint that these peaks are located at the expected nucleosome distance from each other. Our tool PuFFIN (Positioning for Fuzzy and FIxed Nucleosomes) instead uses a novel multi-resolution approach: while its algorithm is relatively simple, our approach introduces some novel ideas that have the potential to be useful in other domains of genome analysis.

## Methods

Our method consists of three steps: (A) we build a set of nucleosome profiles and nucleosome "landscapes"; (B) we detect candidate nucleosome locations on each profile; (C) we select a "consensus" set of nucleosomes that satisfies non-overlapping constraints. We discuss these steps in detail in the next subsections.

### Computing nucleosome profiles and nucleosome landscapes

We first map sequenced reads to the reference genome and then compute a *nucleosome profile *that represents the likelihood that a genomic location is occupied by a nucleosome. Candidate nucleosomes are detected at the peaks of the nucleosome profile. In order to reduce false positives, profiles have to be cleaned from their high frequency component. Choosing the best smoothing method (and its parameters) is, however, not easy. For instance, in [4] the authors show that the kernel density estimation method [12] works significantly better than moving average-based smoothing. The choice of kernel parameters is also important: too much smoothing can merge adjacent peaks, too little can leave noisy artifacts that can be interpreted as peaks and thus introduce spurious nucleosomes. To address the challenges of choosing the "right" kernel and smoothing parameters, we follow an alternative (novel) procedure to construct nucleosome profiles.

First, we replace each mapped paired-end read *i *with a function $f_{i}^{\alpha w_{i}}$ distributed as a Gaussian with mean *µ_i _*and standard deviation *αw_i_*, i.e.,

$$f_{i}^{\alpha w_{i}}\left(x\right)=\frac{1}{\alpha w_{i}\sqrt{2\pi }}e^{-\frac{{\left(x-\mu_{i}\right)}^{2}}{{\left(\alpha w_{i}\right)}^{2}}}$$

where *µ_i _*is the genomic center location of read *i, w_i _*is the length of read *i *(i.e., the distance between the leftmost nucleotide in the left mate and rightmost nucleotide of the right mate), and *α *is a smoothing parameter. Replacing each mapped read with a Gaussian distribution allows us to model probabilistically the uncertainty in the paired-end mapping. For instance, when the left and right mate are mapped far from each other, the mass of the Gaussian will be distributed on a longer interval because of its large variance. If instead the left and right mate are close to each other, the Gaussian will have its mass concentrated at the center of the read, indicating a higher confidence in the nucleosome position.

Then, we compute the *nucleosome profile S_α _*as the weighted sum of functions $f_{i}^{\alpha }$ for all the mapped reads in the input

$$S_{\alpha }\left(x\right)=\overset{n}{\underset{i=1}{\sum }}\beta_{i}f_{i}^{\alpha w_{i}}\left(x\right)$$

where *n *is the number of mapped reads in input, and *β_i _*is the weight of the read *i*. If we had employed a uniform weighting scheme $\left(\beta_{i}=\frac{1}{n}\right)$, paired-end reads with very short insert would dominate the profiles. To reduce the effects of short DNA fragments, we use a non-uniform weighting scheme. For paired-end reads that are shorter than 146bp, we assign a penalty factor *γ*(*w*) *<*1, such that the shorter the read is, the less the weight is (i.e., $\beta_{i}=\frac{1}{n}\gamma \left(w_{i}\right)$). Additionally, one could use the weights *β_i _*to account for sequence quality of individual reads, mappability biases, etc.

As said, parameter *α *controls the smoothness of function *S_α_*. The bigger *α* is, the smoother *S_α _* is, (peaks will be wider), and vice versa. When *α *is large, we capture nucleosome binding preferences at a lower resolution scale; when *α *is small we can detect nucleosomes at a high resolution scale (but noisier). In the limit *α → *0, function $S_{\alpha }\left(x\right)\to \sum_{i=1}^{n}\chi \left(x-\mu_{i}\right)$, where $\chi \left(x\right)=\left\{\begin{array}{c} 1,x=0\\ 0,x\neq 0 \end{array}\right)$ is the indicator function. In this case, *S*_0_(*x*) represents how many read centers cover location *x *in the genome.

One might think that one could obtain the same profiles by computing the read coverage function smoothed by a Gaussian kernel. There is, however, a significant difference: the size of each mapped read *independently *influences the shape of *S_α _*(no matter what smoothing parameter is chosen), while in the case of kernel smoothing the impact of read sizes becomes less and less important as the smoothing strength increases.

Since we do not know the appropriate value for *α *for the data, in this step we generate a family of functions for several choices of *α*. Formally, we create a set of *m *functions ${\left\{S_{\alpha_{k}}\right\}}_{k=1,2,\ldots ,m}=\left\{S_{\alpha_{1}},S_{\alpha_{1}},\ldots ,S_{\alpha_{m}}\right\}$, where *α*_1 _*< α*_2 _*< . . . < α_m _*are *m *distinct choices for *α*. The value *m *is hard-coded in our implementation (we used *m *= 40 for all the experiments).

The set of functions ${\left\{S_{\alpha_{k}}\right\}}_{k=1,2,\ldots ,m}$ enables our algorithm to detect candidate locations for nucleosomes at different resolution scales, thus eliminating the need to specify in advance the parameters for the range of nucleosome-enriched fragments. In other words, our algorithm can "adapt" to the local properties of the input data by processing the same location at different resolutions (corresponding to the choices of *α*).

Finally, we compute a set of *nucleosome landscapes ${\left\{N_{\alpha_{k}}\right\}}_{k=1,2,\ldots ,m}$*by normalizing each function $S_{\alpha_{k}}$ by the lowest resolution function *S_A_*, as follows

$$N_{\alpha_{k}}\left(x\right)=\text{log}\left(\frac{S_{a_{k}}\left(x\right)+\varepsilon }{S_{A}\left(x\right)+\varepsilon }\right)$$

where *ε >*0 is a small constant to avoid a division by zero, and *A >*max_*k *= 1,2*,...,m *_*α_i_*. In our implementation we pick *α *to range from 0.05 to 0.63 and *A *= 1.5. Since mappability biases affect each function $\left\{S_{\alpha_{k}}\right\}$, we can effectively reduce these biases by taking the log ratio of high-resolution and low-resolution function. Another reason to carry out this normalization step is to reduce the differences in the peak heights.

**Figure 1:**
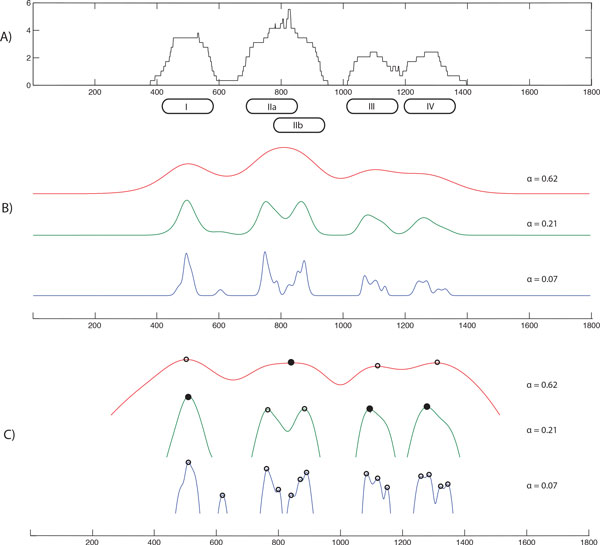
**Synthetic illustrative example**. A) Read coverage; B) Nucleosome profiles $\left\{S_{\alpha_{k}}\right\}$ for *α*_1 _= 0.07 (blue), *α*_2 _= 0.21 (green), *α*_3 _= 0.62 (red) C) Corresponding nucleosome landscapes $\left\{N_{\alpha_{k}}\right\}$ (see text for detailed explanation)

To illustrate the multi-resolution approach in our algorithm, we created a small synthetic dataset with four nucleosomes shown in Figure 1. Panel A shows the raw coverage obtained by mapping synthetic paired-end reads to the reference genome. Observe that nucleosomes I,III and IV are strongly positioned, while nucleosome II is "fuzzy". Fuzzy nucleosomes are quite common and occur when a subset of the cells in the sample has a nucleosome at one location, while in the other subset the same nucleosome is slightly shifted. Nucleosome I is isolated, while nucleosomes III and IV are located very close to each other. Panel B shows the family of functions $\left\{S_{\alpha_{k}}\right\}$ for three choices of *α*; panel C illustrates the set of nucleosome landscape functions $\left\{N_{\alpha_{k}}\right\}$. Observe in Figure 1C that the transformation amplifies candidate peaks in areas with low coverage and reduces the amplitude of peaks in regions with high coverage.

### Detecting candidate nucleosomes

By construction, a nucleosome landscape $N_{\alpha_{k}}$ represents a non-parametric distribution of nucleosomes at resolution *α_k _*. The presence of a peak in any nucleosome landscape indicates a candidate nucleosome. The reads that form corresponding peak belong to that candidate.

A peak is defined by a pair (*q, s*) where *q *is the center of the peak and *s *is the width of the peak. We say that (*q, s*) is a *peak *for function *N *when *N *(*q*) is local maximum for *N *and *s *= min*_z _*(*|q − z|*) where *z *is any local minimum for function *N *.

Detecting peaks on each function $N_{\alpha_{k}}$ can be easily computed in linear time along the length of the genome. As a result, for every choice of *α_k_, k *= 1, 2*, . . . , m *we have a set of peaks $\left\{p_{k_{1}},p_{k_{2}},\ldots ,p_{k_{l}}\right\}$, where $p_{k_{j}}$ is a pair (center, width) representing the peak, and *l *is the number of peaks.

Peaks are however not guaranteed to have a symmetric shape. We therefore recompute the location of every nucleosome candidate as the centroid location of its read midpoints. This additional step ensures that candidate nucleosome locations properly represent the corresponding input reads.

### Building the final solution

We now explain how to build the final set of non-overlapping nucleosomes from the family of peak sets ${\left\{P_{\alpha_{k}}\right\}}_{k=1,\ldots ,m}$. We say that two peaks (*q*_1_*, s*_1_) and (*q*_2_*, s*_2_) *overlap *if *|q*_1 _*− q*_2_*| <*146 (the size of a nucleosome). Observe that by construction, the number of peaks detected at lower resolution (i.e., for large *α*) will be smaller than or equal to the number of peaks detected at higher resolution, i.e., *|P_α_| ≤ |P_β_| *when *α > β*. As we increase the smoothing parameter *α*, the total number of peaks decreases: while some peaks are preserved, others are merged. In other words, for every peak in *P_α _*we can find at least one corresponding peak in *P_β _*if *α > β*.

**Figure 2:**
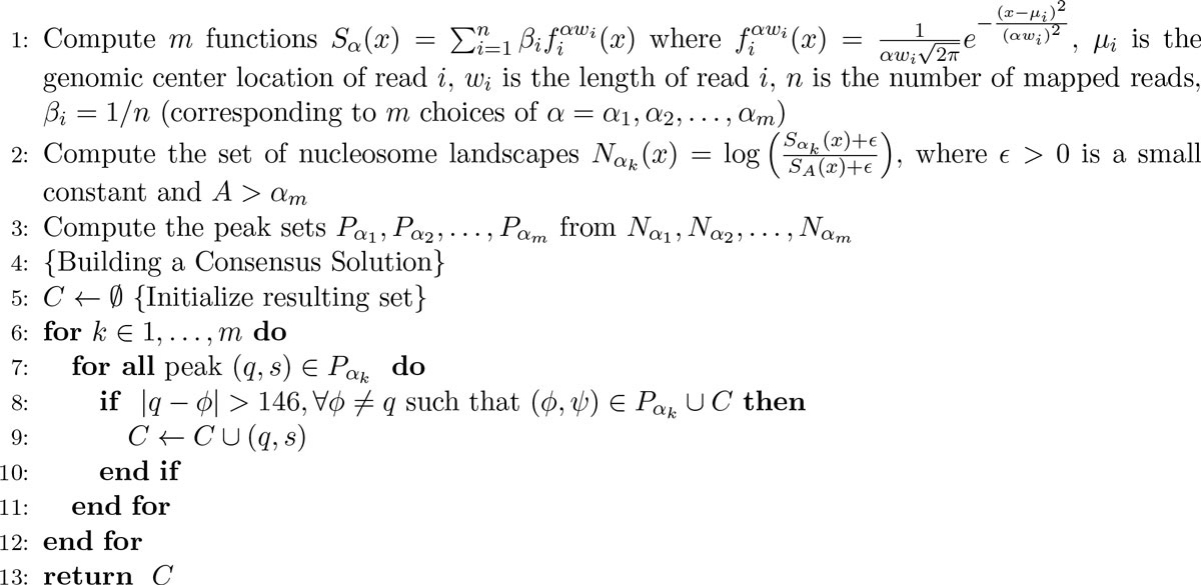
**Sketch of the proposed algorithm**.

Based on this observation, we build the final set of non-overlapping nucleosomes *C *as follows. Given a family of peak sets ${\left\{P_{\alpha_{k}}\right\}}_{k=1,\ldots ,m}$ where *α*_1 _*< α*_2 _*< . . . < α_m_*, we process each peak set $P_{\alpha_{k}}$ in increasing order for *α*. We add a peak *p *from the current set $P_{\alpha_{k}}$ to the final solution *C *if *p *does not overlap with any other peak in the set $P_{\alpha_{k}}$ and if *p *does not overlap with any other peak already in *C*. A sketch of the algorithm can be found in Figure 2.

Let us consider again our example in Figure 1. Detected peaks are marked with circles in panel C. The algorithm first processes the set of peaks on the blue function (*α *= 0.07). Since there are no peaks on that curve that are located at a distance greater than 146bp from each other, the final set *C *remains empty. Next, the algorithm processes the green curve (*α *= 0.21): here there are three peaks that satisfy the non-overlapping constraint. Thus, the algorithm adds those peaks (marked with solid circles) to *C*. Then, the algorithm considers the red curve (*α *= 0.62): all four peaks are non-overlapping with each other, however only one peak (marked with the solid circle) can be added to *C*. As a result, the final solution *C *consists a set of four peaks that match the original nucleosomes. Observe that strongly positioned nucleosomes I, III and IV are detected earlier in the algorithm (*α *= 0.21) than fuzzy nucleosome II (*α *= 0.62).

### Running time

To compute a set of profile functions *S_α _*we use a precomputed set of curves $f_{i}^{\alpha w}$ for every choice *α *and *w *in a predefined range. As a result, it takes Θ(*nm*) operations, where *n *is the number of reads and *m *is the number of curves. In our implementation we used *m *= 40 choices of equally distributed values for *α *∈ [0.05, 0.63].

Finding peaks on each curve *S_α _*takes Θ(*l*) time, where *l *is the length of the processed region. Thus, the total time to find candidate nucleosomes (Figure 2, lines 1-3) is Θ(*m*(*n *+ *l*)). Building the resulting set of non-overlapping nucleosomes is determined by the number of candidates that is at most Θ(*ml*). Given that *m *is predefined, it follows that the total running time is linear in the region size and number of input reads.

## Experimental results

To evaluate the performance of PuFFIN, we performed extensive benchmarking against NucPosSimulator, TEMPLATE FILTERING and NOrMAL. NucPosSimulator is the only published tool designed to deal with paired-end reads [11]. As said, it solves the optimization problem of selecting the subset of peaks which maximizes the total score, under the constraint that these peaks are located at the expected nucleosome distance from each other. TEMPLATE FILTERING is one of the first algorithms developed to infer the size of the fragments from single-end reads [3]. NOrMAL uses a modified Gaussian model to cluster input single-reads such that every cluster represents a nucleosome [6]. Some of the recently published tools that use a control sample to solve the nucleosome positioning problem, e.g., DANPOS and NucleoFinder , are not included in this comparison.

We used default parameters for each tool except for the following provisions. For TEMPLATE FILTERING and NOrMAL we set to zero the allowed overlap between adjacent nucleosomes to allow for a fair comparison with PuFFIN and NucPosSimulator.

Arguably the major challenge for nucleosome position inference is that the true positions of nucleosomes are unknown. The lack of a "ground-truth" makes it very hard to benchmark existing computational methods. For this reason we made extensive use of synthetic data, as explained next.

### Results on synthetic data

**Figure 3:**
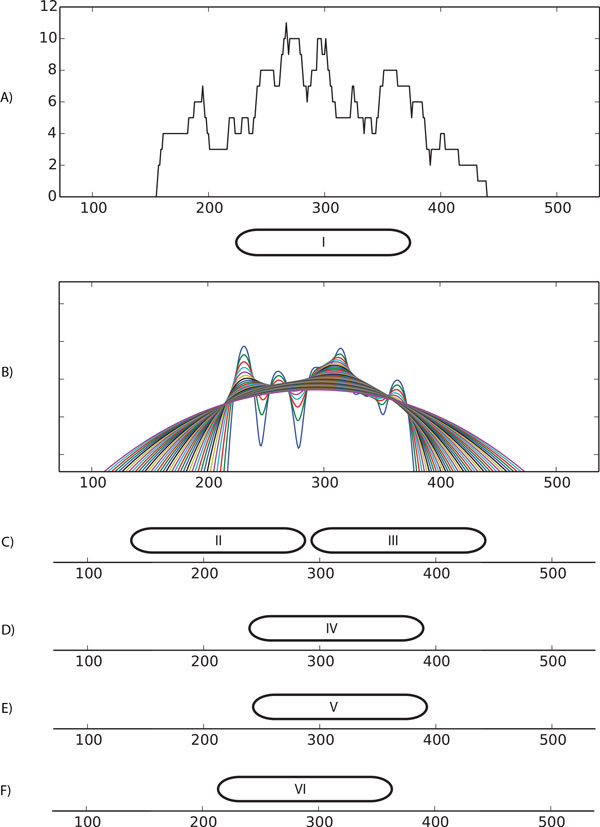
**A "toy" example**. A) Raw coverage; B) Nucleosome landscapes for different choices of *α *∈ [0.05, 0.63); C) NucPosSimulator result; D) PuFFIN result; E) TEMPLATE FILTERING result; F) NOrMAL result;

We started by producing a small dataset of reads corresponding to DNA-enriched fragments for only one nucleosome (Figure 3). This allowed us to investigate the behavior of these various tools in the scenario of low sequence coverage in a region containing a fuzzy nucleosome. Nucleosome I is centered at 300bp and the paired-end reads of size 146bp were generated with midpoints distributed according to Gaussian with mean 300, and standard deviation 40. To simulate a low coverage scenario, we generated only twenty sequence reads (20-fold coverage). PuFFIN, TEMPLATE FILTERING and NOrMAL report one nucleosome located at 308bp, 311bp and 292bp, respectively, while NucPosSimulator reports two nucleosomes positioned at 221bp and 369bp. The slight difference of the reported locations for the first three tools could be explained by the small sample size that is insufficient to recover the true location. Interestingly, the first two methods, which are based on peak-detection, produced a similar close right shift, while the nucleosome detected by NOrMAL showed a small left shift. NucPosSimulator detected two distinct nucleosomes, probably because the objective of this tool is to maximize the total score of reported nucleosomes. We believe that maximizing this quantity has the undesirable effect to over-report nucleosomes (i.e., increase false positives). Decreasing the smoothing parameter in NucPosSimulator from 20.0 (default) to 2.0 reduces the output to a single nucleosome, again demonstrating how the choice of smoothing parameters can have significant effects on the results.

Next, we performed a more realistic comparison on *in silico *reads for larger synthetic nucleosome maps. We used the nucleosome map generator syntheticNucMap from nucleR [4]. This tool allows users to specify the number of well-positioned and fuzzy nucleosomes, as well as the variance for the location of synthetic reads and the coverage level. Well-positioned nucleosomes are placed along the chromosome regularly spaced with a fixed linker size (we used linkers of 20bp, which introduces a periodicity of *≈ *167bp). For fuzzy nucleosomes, locations are picked at random and independently from other nucleosomes already on the chromosome. As a consequence, fuzzy nucleosomes can overlap with other nucleosomes. For the variance parameter we choose 30 bases for well-positioned and 50 bases for fuzzy nucleosomes.

**Figure 4:**
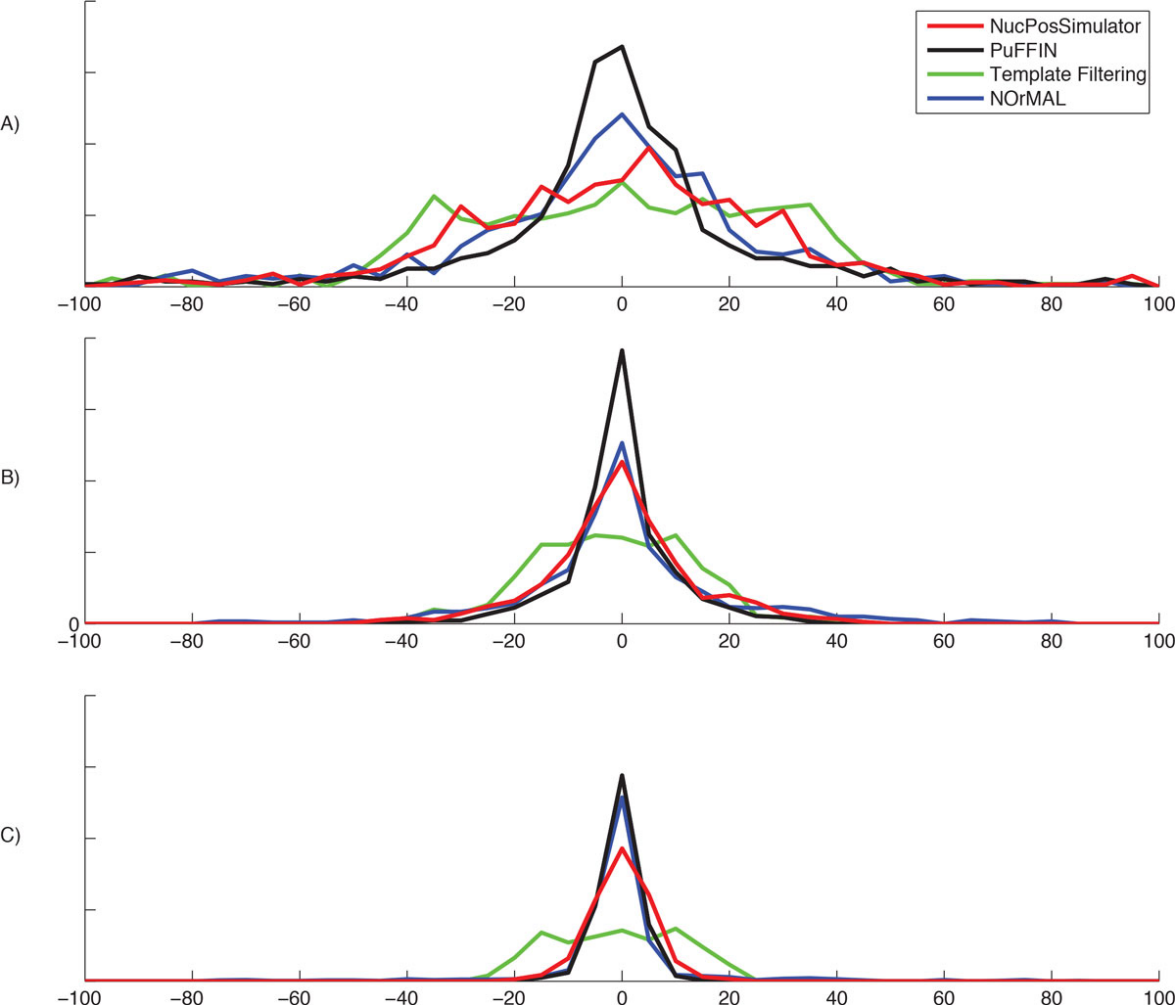
**Distribution of the distances between detected and true locations for A) 100%, B) 60% and C) 0% fuzzy dataset**.

**Figure 5:**
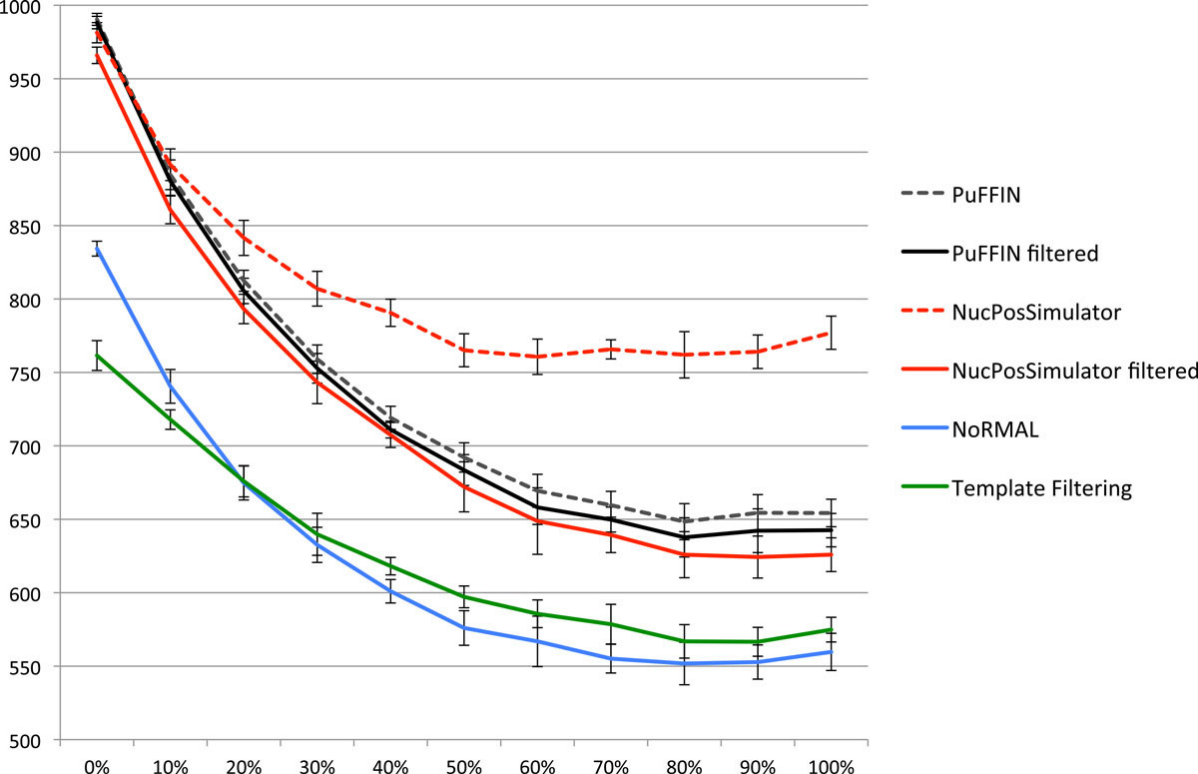
**Dependency between the percentage of fuzzy reads in the sample (X axis) and the number of detected nucleosomes (Y axis) for synthetic dataset**.

**Figure 6:**
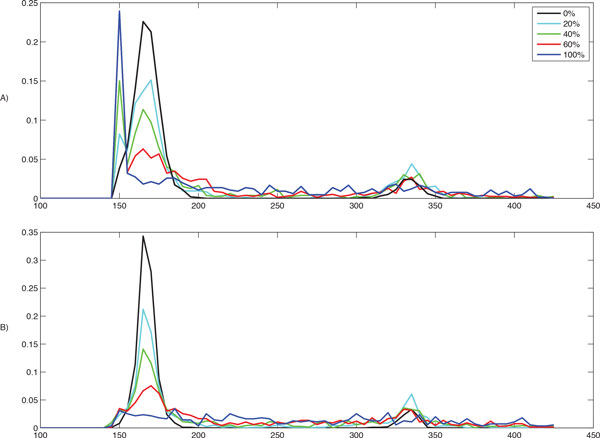
**Distribution of the distances between adjacent nucleosomes for A) NucPosSimulator B) PuFFIN**.

Our objective was to investigate the accuracy of nucleosome detection as a function of the fraction of fuzzy nucleosomes: we expected the detection problem to become increasingly harder as the number of fuzzy/overlapping nucleosomes increases. For each percentage level of fuzzy nucleosomes (0%, 10% . . . , 100%) we generated ten datasets of synthetic reads for a map containing 1,000 synthetic nucleosomes. To build these datasets, we used the following command: syntheticNucMap(wp.num = 1100, wp.del=(100+r*100), wp.var = 30, fuz.num=(r*100), fuz.var = 50, max.cover = 70, nuc.len = 147, lin.len = 20), where *r *controls the fraction of fuzzy nucleosomes (*r *= 0 is 0%, *r *= 1 is 10%, . . . , *r *= 10 is 100%). For each group of ten datasets we measured the number of reported nucleosomes and the accuracy of each tool, and reported the average and standard deviation over the ten sets. To measure the accuracy, we calculated the distances between the true nucleosome location and the center of the corresponding detected nucleosome. Results in Figure 4 show that PuFFIN reports nucleosome positions more accurately in datasets with larger proportions of fuzzy nucleosomes. In addition, Figure 5 shows the average number of nucleosomes detected by the various tools for increasing percentages of fuzzy nucleosome (the error bar represents the standard deviation over the ten datasets). First observe that although each dataset is expected to have synthetic reads for exactly 1,000 nucleosomes, this is only true for datasets with no fuzzy nucleosomes. Since fuzzy nucleosomes may overlap other nucleosomes, we expect to detect a decreasing numbers of nucleosomes as the percentage of fuzzy nucleosomes increases (which is reflected in Figure 5). Also observe in Figure 5 that in datasets with more than 20% of fuzzy nucleosomes, NucPosSimulator detects the highest number of nucleosomes compared to other tools. However, as we demonstrated earlier in Figure 3, NucPosSimulator can over-report nucleosomes. To explore whether this was true on these larger datasets, we computed the distribution of distances between adjacent nucleosomes (Figure 6). In the group of datasets with no fuzzy nucleosomes, both NucPosSimulator and PuFFIN have strong peak at around 167bp location and 334bp. This is expected, because all nucleosomes are well-positioned and are located at multiples of 167bp. However, as we increase the percentage of fuzzy nucleosomes in the datasets, NucPosSimulator reports more and more nucleosomes exactly 148 bp apart from each other, which suggests that its strategy to maximize the total score for reported nucleosomes has the effect of reporting too many nucleosomes.

To eliminate the effects of over-reporting in NucPosSimulator, we discarded from the counts nucleosomes that are located 148 bases or less from each other, such that every pair of tightly placed nucleosomes is count as one nucleosome. In Figure 5, curves marked "filtered" shows the results of this cleaning step. Observe that the number of nucleosomes reported by NucPosSimulator drops significantly, while only a small number of PuFFIN nucleosomes are affected. In fact, using this cleaning step, PuFFIN reports a larger numbers of nucleosomes than NucPosSimulator. All together, these experimental results on synthetic data show that PuFFIN generates more accurate nucleosome maps, without over-reporting nucleosomes.

### Results on real data

For the comparison of nucleosome positioning tools, we used a publicly available dataset for *S. cerevisiae *(NCBI SRA SRR094649) and our dataset for *P. falciparum *(NCBI SRA SRS453761). All datasets contain paired-end reads produced by an Illumina sequencing instrument. Reads were mapped to their corresponding reference genomes using Bowtie2 [13] with --very-fast-local --no-discordant flags. We removed reads that were not mapped uniquely or had a distance between the left and right mates smaller than 40bp or bigger than 1,000bp.

Experimental results are summarized in Table 1, which include the number of reported nucleosomes and the execution time. Nucleosome positioning in *S. cerevisiae *is extensively studied and the majority of the tools perform well on this organism. Also, nucleosomes in yeast are well-positioned and not many overlaps are present. The results in Table 1 show that the number of nucleosome reported in yeast by these tools are quite similar, except for NucPosSimulator that reports a significantly larger number. These results possibly again suggest the over-reporting behavior of this tool.

**Table 1:** Number of reported nucleosomes and execution times on yeast and the human malaria parasite.

	*S. cerevisiae *(W303 contig 7)		*P. falciparum *(3D7 chr. 2)	
	**# Nucleosomes**	**Time (sec)**	**# Nucleosomes**	**Time (sec)**

TEMPLATE FILTERING	630	**1**	2,725	**13**

NOrMAL	592	4	3,247	40

NucPosSimulator	**802**	75	3,722	920

PuFFIN	709	165	**3,760**	350

Our previous work [6] has demonstrated that the *P. falciparum *genome has a greater complexity of nucleosomes configurations. As expected, experimental results show much greater variance in the number of nucleosomes in the malaria dataset reported by the various tools. PuFFIN reports a similar number of nucleosomes compared to NucPosSimulator, but significantly higher numbers than NOrMAL and TEMPLATE FILTERING, indicating that our method is capable to resolve complex configurations of nucleosomes.

The execution time of PuFFIN is higher than NOrMAL and TEMPLATE FILTERING on both datasets, but shorter than NucPosSimulator on *P. falciparum *and higher on *S. cerevisiae *datasets. Our implementation of PuFFIN is currently written in Python, while the other tools use either Java or C/C++. We believe that speed of our tool could be easily improved by one order of magnitude by implementing it in C/C++ (work in progress).

**Figure 7:**
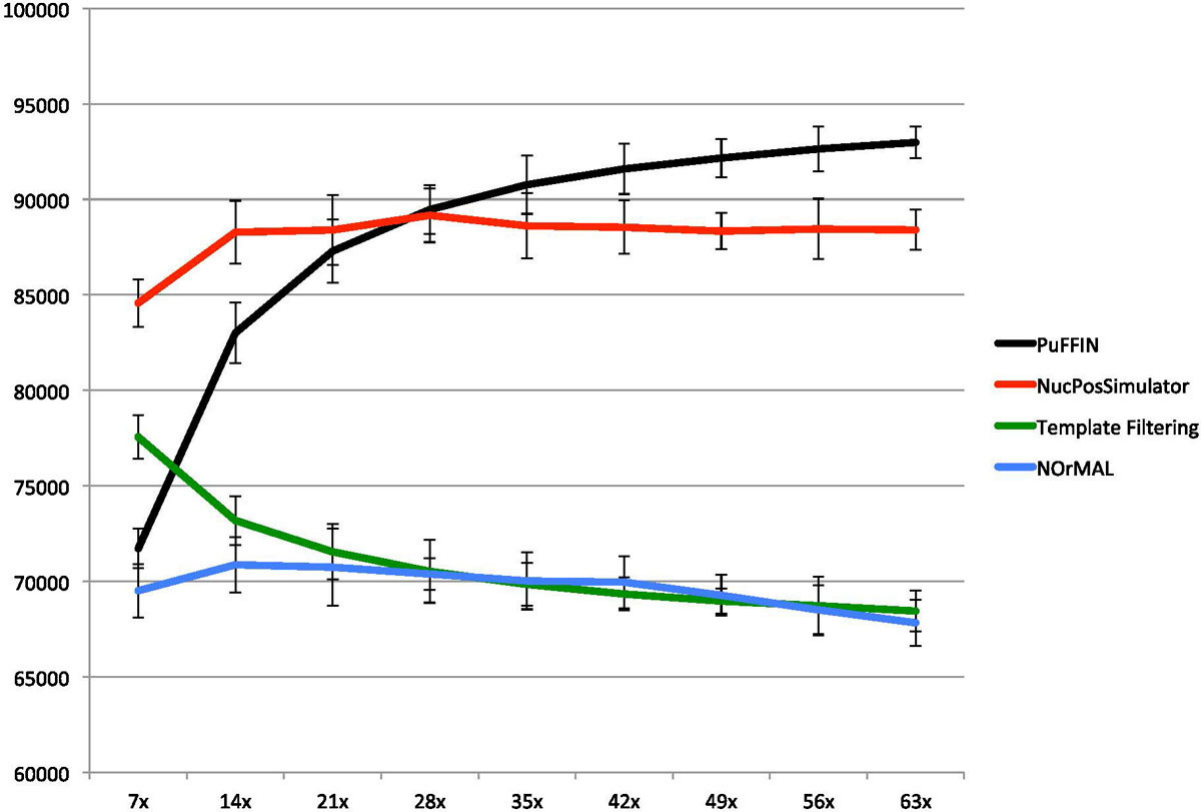
**Dependency between the fold coverage (X axis) and number of detected nucleosomes (Y axis) for *P. falciparum***.

To investigate the sensitivity of the tools on the quantity of the input data (coverage), we performed an experiment in which an increasing fractions of the input reads were discarded. Specifically, we sampled the *P. falciparum *dataset by randomly selecting a given fraction of the input reads (20%, 30% . . . , 100%) and ran the four tools on the resulting datasets. Subsamples have 7x, 14x,. . . , 63x fold coverage. Figure 7 shows that the performance of PuFFIN degrades monotonically as the quantity of the data decreases, while NucPosSimulator remains more stable over a larger range of input data. We therefore recommend to use sequence data with a minimum of 30-fold for the analysis of nucleosome positions if PuFFIN is used.

## Conclusion

We described a novel method to solve the nucleosome positioning problem when paired-end data is available. Our method employs a multi-resolution strategy that circumvents a smoothing step that usually requires user-defined parameters to set the strength of the smoothing and type of kernel to be used. Experimental results show that our method more accurately detects nucleosome positions as compared to existing software tools, in particular when complex nucleosome configurations are present in the data.

## Competing interests

The authors declare that they have no competing interests.

## Authors' contributions

KLR and SL conceived and supervised the project; AP designed, implemented, tested and debugged PuFFIN; EMB generated experimental nucleosome positioning datasets in *P. falciparum*; AP performed all computational analyses; AP, SL, and EMB wrote the manuscript. All authors read and approved the final manuscript.

## Declarations

The authors declare that funding for publication of this article was sponsored by National Science Foundation, grant IIS-1302134.

This article has been published as part of BMC Bioinformatics Volume 15 Supplement 9, 2014: Proceedings of the Fourth Annual RECOMB Satellite Workshop on Massively Parallel Sequencing (RECOMB-Seq 2014). The full contents of the supplement are available online at http://www.biomedcentral.com/bmcbioinformatics/supplements/15/S9.
